# Nesfatin-1-Like Peptide Encoded in Nucleobindin-1 in Goldfish is a Novel Anorexigen Modulated by Sex Steroids, Macronutrients and Daily Rhythm

**DOI:** 10.1038/srep28377

**Published:** 2016-06-22

**Authors:** Lakshminarasimhan Sundarrajan, Ayelén Melisa Blanco, Juan Ignacio Bertucci, Naresh Ramesh, Luis Fabián Canosa, Suraj Unniappan

**Affiliations:** 1 Laboratory of Integrative Neuroendocrinology, Department of Veterinary Biomedical Sciences, Western College of Veterinary Medicine, University of Saskatchewan, Saskatoon, Saskatchewan S7N 5B4, Canada; 2 Departamento de Fisiología (Fisiología Animal II), Facultad de Biología, Universidad Complutense de Madrid, 28040 Madrid, Spain; 3 Instituto de Investigaciones Biotecnológicas-Instituto Tecnológico Chascomús (IIB-INTECH), Intendente Marino Km 8.2, B7130IWA Chascomús, Buenos Aires CC 164 (7130), Argentina

## Abstract

Nesfatin-1 is an 82 amino acid anorexigen encoded in a secreted precursor nucleobindin-2 (NUCB2). NUCB2 was named so due to its high sequence similarity with nucleobindin-1 (NUCB1). It was recently reported that NUCB1 encodes an insulinotropic nesfatin-1-like peptide (NLP) in mice. Here, we aimed to characterize NLP in fish. RT- qPCR showed NUCB1 expression in both central and peripheral tissues. Western blot analysis and/or fluorescence immunohistochemistry determined NUCB1/NLP in the brain, pituitary, testis, ovary and gut of goldfish. NUCB1 mRNA expression in goldfish pituitary and gut displayed a daily rhythmic pattern of expression. Pituitary NUCB1 mRNA expression was downregulated by estradiol, while testosterone upregulated its expression in female goldfish brain. High carbohydrate and fat suppressed NUCB1 mRNA expression in the brain and gut. Intraperitoneal injection of synthetic rat NLP and goldfish NLP at 10 and 100 ng/g body weight doses caused potent inhibition of food intake in goldfish. NLP injection also downregulated the expression of mRNAs encoding orexigens, preproghrelin and orexin-A, and upregulated anorexigen cocaine and amphetamine regulated transcript mRNA in goldfish brain. Collectively, these results provide the first set of results supporting the anorectic action of NLP, and the regulation of tissue specific expression of goldfish NUCB1.

Nucleobindins (NUCB1 and NUCB2) are a class of multi-domain Ca^2+^ and DNA binding proteins that play an important role in cell signaling^1^. Nucleobindins are multifunctional proteins and are proposed as precursors of bioactive endocrine regulatory factors^1^. Human NUCB1 and NUCB2 are remarkably conserved (62% amino acid identity) within their bioactive regions (24–53 amino acids)^2^,^3^,^4^. In fact, NUCB2 was named so due to its high sequence similarity with NUCB1. In 2006, a novel anorexigen named nesfatin-1 (NEFA/nucleobindin-2-Encoded Satiety and Fat-Influencing proteiN-1), an 82 amino acid anorexigenic peptide encoded in the N-terminal region of nucleobindin-2 (NUCB2) was reported^5^. NUCB2 is cleaved by prohormone convertases (PC 1/3 and 2) resulting in three peptide fragments, nesfatin-1, nesfatin-2 and nesfatin-3; of which nesfatin-1 is the only one known to be biologically active^5^. Administration of the bioactive core (M30, mid-segment 30 amino acids) of nesfatin-1 inhibits food intake and reduces body weight in rodents^5^,^6^. In rats, nesfatin-1 inhibits feeding and promotes energy expenditure^7^. In mice, nesfatin-1 secretion is modulated by nutrients suggesting that nesfatin-1 plays an important role in metabolism and energy homeostasis^8^. Administration of nesfatin-1 (25 pmol/rat) affects thermogenesis, resulting in stimulation of energy expenditure and lowering of food intake in rats^9^. In goldfish, nesfatin-1 reduces food intake^10^,^11^ and reproductive hormone secretion^10^,^11^,^12^. Nesfatin-1 was also detected in zebrafish^13^, Ya fish^14^ and trout^15^. Nesfatin-1 is now considered a multifunctional peptide in fish and mammals.

More recently, NUCB1 gained attention due to its similarity with NUCB2 and nesfatin-1. For example, our *in silico* analysis found that NUCB1 in fish and mammals encode a nesfatin-1 like sequence^1^,^16^, and these peptides possess prohormone convertase sites that enable its processing^16^. Immunofluorescence studies also revealed that the localization of NUCB1 is highly concentrated in islet cells in mice^16^. NUCB1 is very highly conserved in mammals and non-mammals. Our lab, for the first time, reported the discovery of a nesfatin-1 like peptide (NLP) in mice and its insulinotropic actions on mice pancreatic beta cells^16^. Whether NLP has appetite regulatory roles remain unknown.

This research aimed to determine two important aspects of NUCB1/NLP in goldfish, a well-characterized model in neuroendocrinology research. The first topic addressed was the tissue specific expression, and regulation of endogenous NUCB1 in goldfish. Second, we determined whether NLP has any effects on food intake in fish. Our results show tissue abundance and cell specific expression of NUCB1/NLP. This research also provides the novel evidence for daily rhythmic pattern under light:dark cycle, steroid, energy status and macronutrient modulation of NUCB1 mRNA expression in goldfish. Finally, we report the discovery of an anorexigenic activity for NLP.

## Results

### *In Silico* Analysis of NUCB1 Sequences

**Figure 1:**
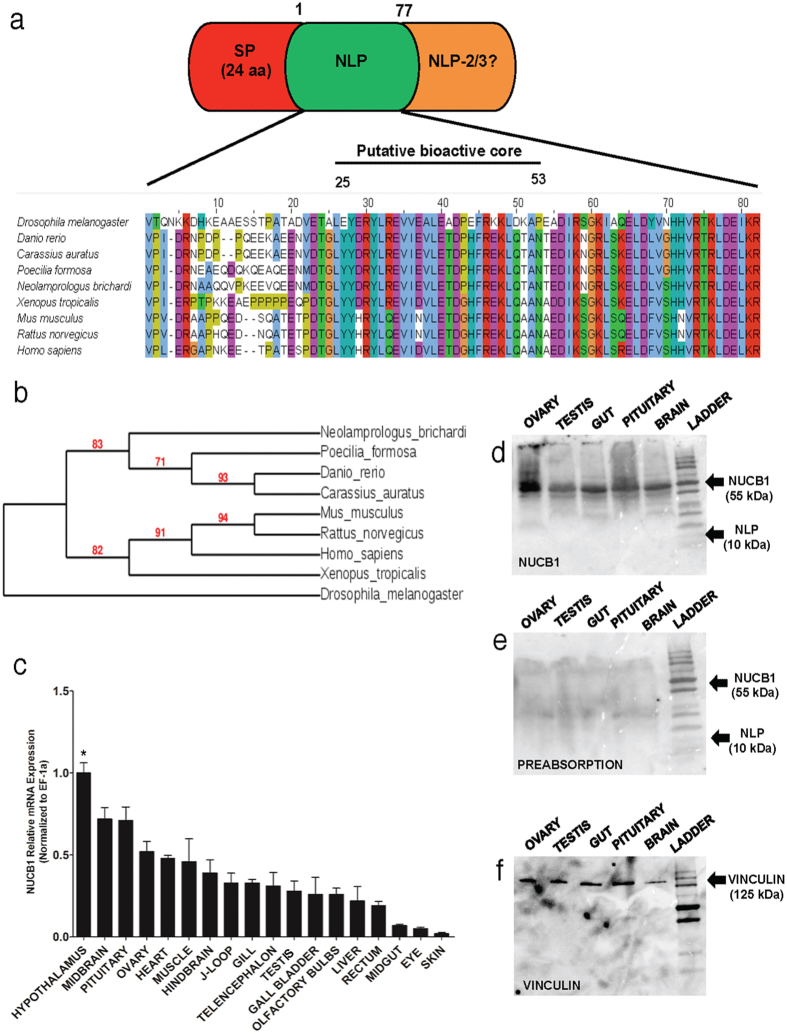
(**a**) Schematic representation of the NUCB1 precursor showing the signal peptide, and nesfatin-1-like peptide (1–77 amino acids) regions. Nesfatin-1-Like Peptide. 2/3 is referred as NLP 2/3. The alignment of NLP sequences from various species is shown underneath. Phylogenetic analysis of nucleobindin-1 gene sequences of various species is shown in **(b)**. NUCB1 sequences consisting of signal peptide (1–24 amino acids) and the putative bioactive core (24–53 amino acids) were used for generating the cladogram. GenBank accession numbers of sequences used are: *Carassius auratus* (KU903286), Neolamprologus brichardi* (XM_006803054.1), *Drosophila melanogaster* (NM_140751.4), Poecilia formosa* (XM_007562129.1), *Xenopus tropicalis* (NM_213689.2) *Danio rerio* (NM_001045463.1), *Mus musculus* (NM_001163662.1), *Rattus norvegicus* (NM_053463.1), *Homo sapiens* (NM_006184.5). Asterisk (*) associated with species names denotes predicted NUCB1 sequences. **(c)** The data was quantified using RT-qPCR (Real-Time - Quantitative PCR) in goldfish. The mRNA expression was normalized to elongation factor (EF)-1α (n = 6 goldfish). Western blot showing NUCB1 in brain, pituitary, gut, ovary and testis **(d)**, preabsorption control showing no immunoreactivity in tissues tested **(e)**, and vinculin **(f)** (n = 4 goldfish, representative blot is shown). Asterisk denotes significant differences (p < 0.05). Data are represented as mean + SEM. One-way ANOVA (non-parametric) followed by Tukey’s multiple comparison test were used for statistical analysis.

Sequence analysis found a very highly conserved nesfatin-1 like peptide (Fig. 1a) encoded in goldfish NUCB1 (GenBank Accession # KU903286). Goldfish NLP is identical to zebrafish NLP (Fig. 1a). The proposed bioactive core (M30) of NLP (77 amino acids) is very highly conserved across species. Goldfish/zebrafish NLP exhibits 74% amino acid sequence identity with zebrafish/goldfish nesfatin-1. A signal peptide cleavage site was predicted at positions 19 (Arginine) and 20 (Valine) in zebrafish and goldfish NLP sequences. Phylogenetic analysis found clustering of goldfish NUCB1 with NUCB1 from other fishes (Fig. 1b).

### Tissue Distribution of NUCB1 in Goldfish

Abundance of NUCB1 mRNA expression was detected in several tissues including the hypothalamus, midbrain, hindbrain, muscle, pituitary, heart, olfactory bulbs and ovary (n = 6 goldfish). The expression of NUCB1 mRNA was normalized to EF-1α, which served as a reference gene to verify the quality and amount of goldfish mRNA samples (Fig. 1c). Western blot analysis detected NUCB1 at 55 kDa in goldfish tissue samples (Fig. 1d). No bands of expected size representing NUCB1 or NLP were detected in the pre-absorption control (Fig. 1e). Vinculin (125 kDa) was used as a reference protein (Fig. 1f). The NUCB1 antibody used here only detected NLP, but not nesfatin-1 (Supplementary Fig. S1). In addition, pre-absorption of this antibody using synthetic goldfish/zebrafish NLP resulted in no staining for NLP (Supplementary Fig. S1).

### Cell Specific NUCB1/NLP-like Immunoreactivity in Gut, Testis and Ovary and Pituitary

**Figure 2:**
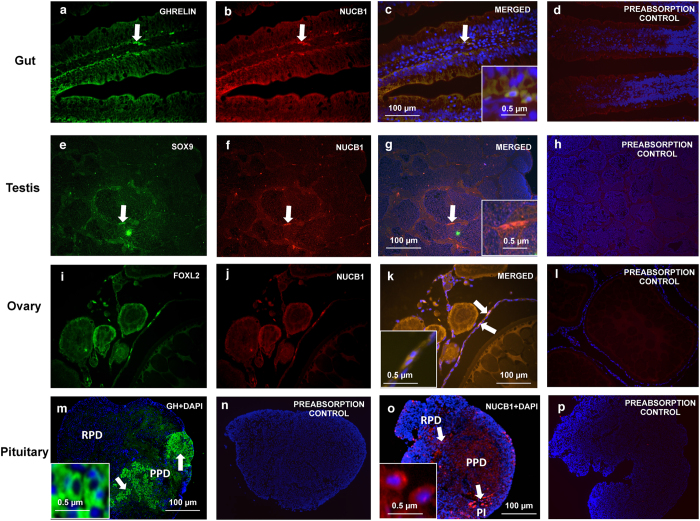
Immunofluorescence representing NUCB1/NLP immunreactivity (Red; Texas Red; and in Green are Ghrelin (J-Loop); SOX9 (Testis); FOXL2 (Ovary); Growth Hormone (Pituitary) in the J-Loop **(a–c)**, testis **(e–g)** and ovary **(i–k)** and pituitary **(m–p)** of goldfish. Nuclei are stained blue (DAPI). Representative cells showing immunoreactivity in goldfish tissues are marked with arrows. A magnified image of representative NUCB1/NLP-ir cell is shown in square inset in figure (**c,g,k,o**) and GH (**n**). Images were taken at 40X magnification and scale bar = 100 μm (and 0.5 μm for inset). No immunoreactivity was detected in GH preabsorption control in goldfish pituitary **(n)**. Similarly, no immunoreactivity was detected in our NUCB1/NLP preabsorption controls **(d,h,l,p)**.

NUCB1/NLP-like immunoreactivity (red) was found in goldfish J-Loop (Fig. 2b), testis (Fig. 2f), ovary (Fig. 2j) and rostral pars distalis (RPD) region of pituitary (Fig. 2o). Ghrelin (Fig. 2a), SOX9 (Fig. 2e), FOXL2 (Fig. 2i) and growth hormone (Fig. 2m) were used as cell specific markers. In the gut, cells in the villi were positive for both NUCB1/NLP and ghrelin (Fig. 2c). Leydig cell cytoplasm in goldfish testis co-localized NUCB1/NLP and SOX9 (Fig. 2g). Thecal and/or follicular cells within the goldfish ovary were stained positive for NUCB1/NLP and FOXL2 (Fig. 2k). DAPI (blue) stained the nuclei of cells. No immunoreactivity was observed in preabsorption controls (Fig. 2n**–**GH,d,h,l,p – NUCB1).

### High Carbohydrate and High Fat Diet Decrease NUCB1 mRNA Expression in Goldfish

**Figure 3:**
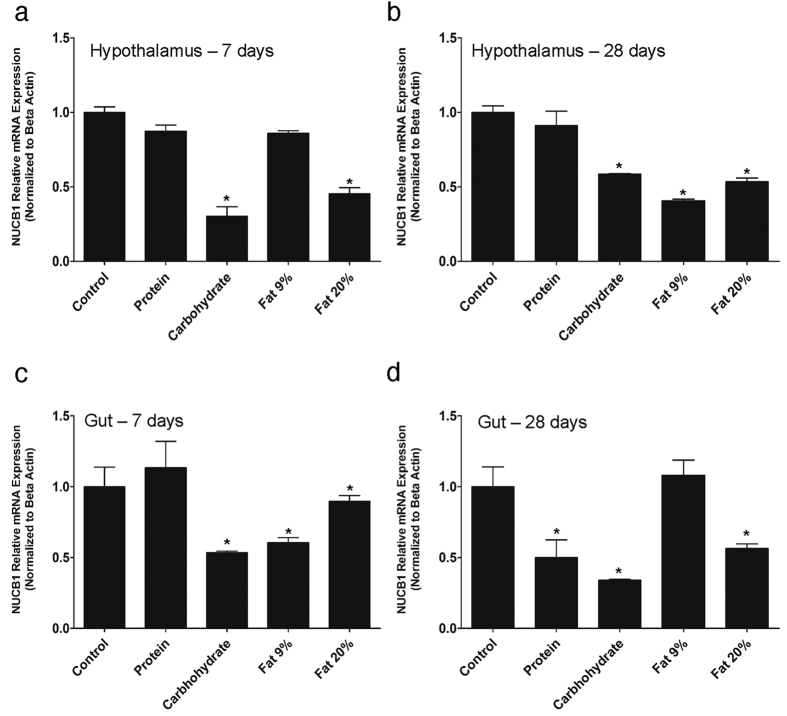
NUCB1 mRNA expression in goldfish hypothalamus after 7 days **(a)** or 28 days **(b)** of feeding special diets containing varying amounts of macronutrients. NUCB1 mRNA expression in goldfish hypothalamus was downregulated in response to high carbohydrate and very high fat (20%) feeding for 7 days **(a)**. NUCB1 mRNA expression was significantly reduced in goldfish hypothalamus post- high carbohydrate and high fat (9% and 20%) feeding for 28 days **(b)**. In gut, NUCB1 mRNA expression was attenuated after high carbohydrate and fat (9%, 20%) after 7 days **(c)**. Meanwhile, NUCB1 mRNA expression was decreased in the gut of goldfish fed on high protein, high carbohydrate and fat (20%) diet after 28 days of feeding **(d)**. NUCB1 mRNA expression were normalized to beta-actin. Asterisks denote significant differences between treatment groups (p < 0.05, n = 8 fish/group). Data are represented as mean + SEM. One-way ANOVA (non-parametric) followed by Tukey’s multiple comparison test were used for statistical analysis.

Seven day feeding of high carbohydrate and very high fat (20%) significantly reduced NUCB1 mRNA expression in the hypothalamus (Fig. 3a). No changes were elicited by high protein and 9% fat diet. Goldfish fed on high carbohydrate, high fat (9%) and very high fat diet (20%) for 28 days had significant attenuation in NUCB1 mRNA expression in the hypothalamus (Fig. 3b). Similar to 7 day results, protein feeding did not cause any changes in NUCB1 mRNA expression in the hypothalamus (Fig. 3b). High carbohydrate and high fat (9% and 20%) feeding for 7 days downregulated NUCB1 mRNA expression in the gut (Fig. 3c). High protein diet feeding for 7 days did not elicit any changes in gut, but decreased NUCB1 expression at 28 days (Fig. 3c,d). Both high carbohydrate and very high fat also caused a similar decrease in NUCB1 mRNA in the gut after 28 days of feeding (Fig. 3d).

### NUCB1 mRNA Expression in Gut and Pituitary Follows a Daily Rhythm Profile

**Figure 4:**
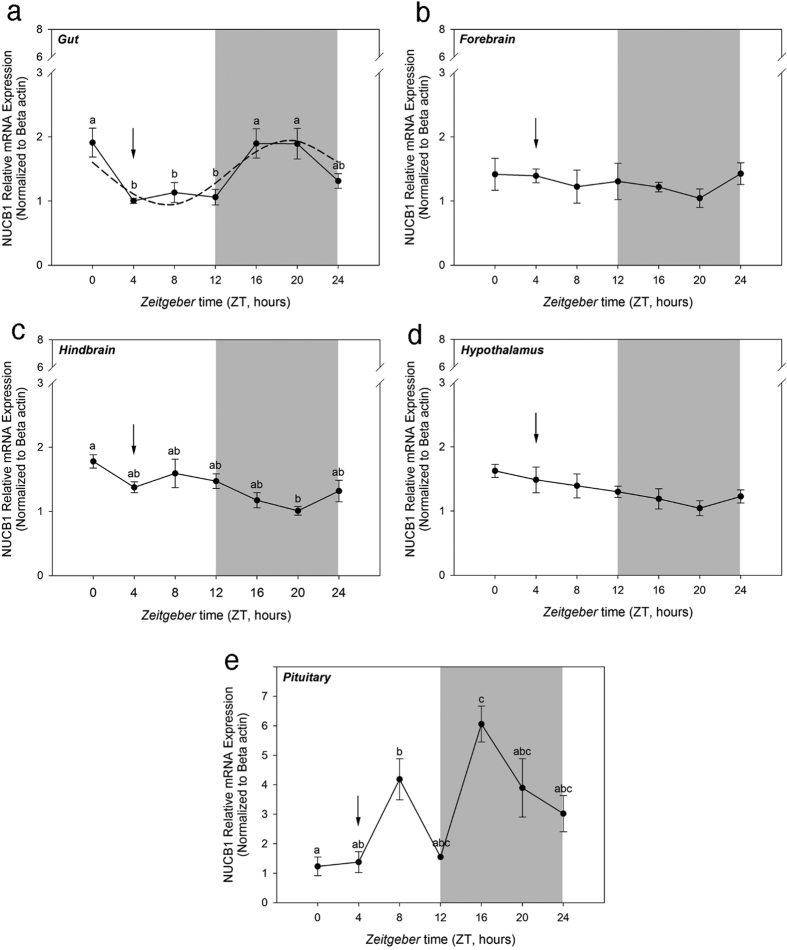
NUCB1 mRNA expression displayed a rhythmic pattern in the gut and pituitary of goldfish. Expression of NUCB1 mRNA expression in the gut **(a)**, forebrain **(b)**, hindbrain **(c)**, hypothalamus **(d)** and pituitary **(e)** of goldfish maintained under 12L: 12D photoperiod and scheduled feeding. The mRNA expression data was normalized to beta-actin. Data are presented as mean + SEM. The grey areas represent the night time and arrows indicate feeding time. Different alphabets denote significant differences between the time points (P < 0.05, n = 6 fish/group). One-way ANOVA (non-parametric) followed by Tukey’s multiple comparison test was used for statistical analysis. The dashed line in figure b indicates a significant rhythm determined by cosinor analysis.

Goldfish maintained under a 12L:12D photoperiod and scheduled feeding at ZT-4 exhibited a rhythmical expression of NUCB1 mRNAs in the gut, with a significant increase during the dark phase of the cycle (ZT-16) that remained until feeding time (Fig. 4a). NUCB1 mRNA expression was observed to be rhythmic also in the pituitary, exhibiting a daily expression profile characterized by one peak during the light and another during the dark phase of the daily rhythmic cycle (Fig. 4e). No daily rhythms in NUCB1 mRNA expression were detected in forebrain (Fig. 4b), hindbrain (Fig. 4c) and hypothalamus (Fig. 4d).

### Testosterone and Estradiol Modulate NUCB1 mRNA in Goldfish

**Figure 5:**
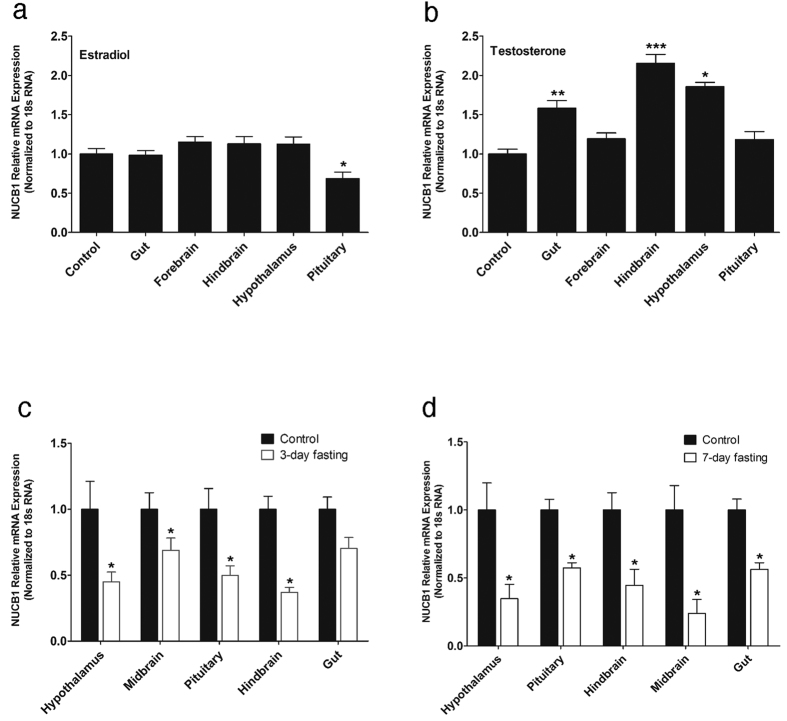
Estradiol (100 μg/g BW) decreased NUCB1 mRNA expression in female goldfish pituitary **(a)**. Testosterone (100 μg/g BW) increased NUCB1 mRNA expression in the gut, hindbrain, and hypothalamus of female goldfish **(b)**. Expression of NUCB1 in gut, forebrain, hindbrain, hypothalamus and pituitary of fish treated with estradiol **(a)** or testosterone **(b)** were normalized to control group. The mRNA expression data were normalized to 18 s RNA. Asterisks denote significant differences between the experimental groups. (*p < 0.05, **p < 0.01, ***p < 0.005, n = 7 female goldfish/group). Food deprivation decreased relative mRNA expression of NUCB1 in goldfish tissues after 3 days **(c)** and 7 days of food deprivation **(d)**. The mRNA expression data were normalized to 18 s RNA. Asterisks denote significant differences between control and fasted groups (*p < 0.05, n = 6 fish/group). Data is represented as mean + SEM. One-way ANOVA (non-parametric) followed by Tukey’s multiple comparison test and Student-Newman-Keuls test **(a, b)** were used for statistical analysis.

NUCB1 mRNA expression was downregulated in the pituitary of goldfish that received estradiol (100 μg/g body weight) (Fig. 5a). NUCB1 mRNA expression was upregulated in gut, hindbrain and hypothalamus of testosterone (100 μg/g body weight) treated goldfish (Fig. 5b). Estradiol and testosterone caused no effect on NUCB1 mRNA expression in other tissues (Fig. 5a,b).

### Food Deprivation Suppresses NUCB1 mRNA Expression

A significant decrease in NUCB1 mRNA expression was observed in hypothalamus, midbrain, pituitary and hindbrain in response to food deprivation for 3 days (Fig. 5c). No change in gut NUCB1 mRNA was detected after 3 days of food deprivation. NUCB1 expression was significantly decreased in both brain and gut after 7 days of food deprivation (Fig. 5d).

### NLP Reduces Food Intake in Goldfish

**Figure 6:**
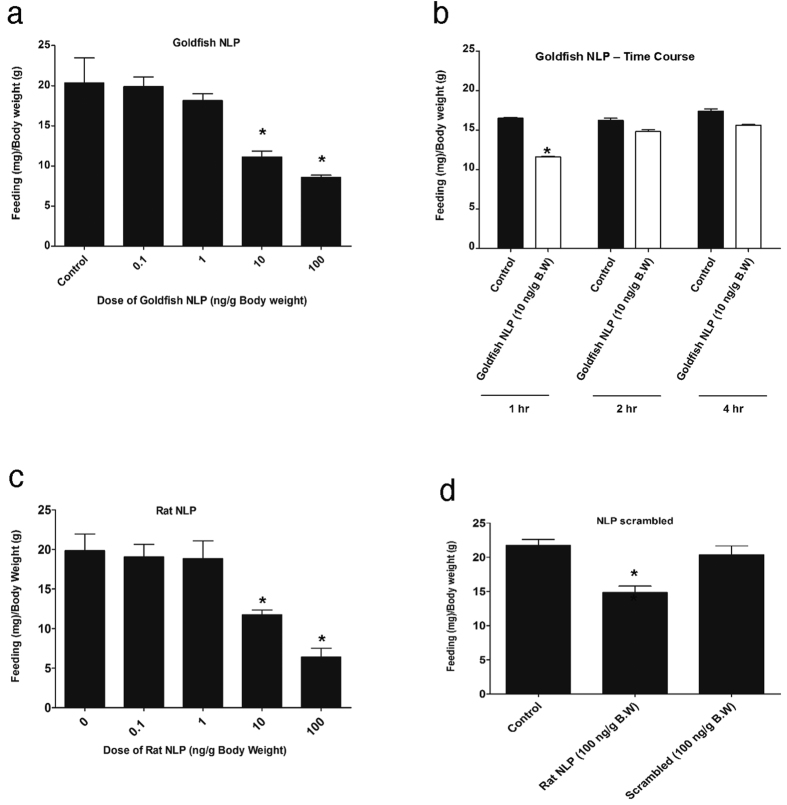
Intraperitoneal injection of synthetic goldfish NLP **(a)** and rat NLP **(c)** reduced food intake when compared to saline injected (control, denoted by 0) samples of goldfish. Also i.p injection of goldfish NLP reduced feed intake during the first hour but recovered after 2 hr and 4 hr post injection when compared to saline injected (control) samples of goldfish **(b)**. However, the NLP based scrambled peptide did not reduce food intake of goldfish **(d)**. Asterisks denote significant differences between control and NLP injected groups of the same (*p < 0.05, n = 10 fish/group). Data are represented as mean + SEM. One-way ANOVA (non-parametric) followed by Tukey’s multiple comparison test were used for statistical analysis.

Administration of 10 and 100 ng/g body weight goldfish NLP (Fig. 6a) and rat NLP (Fig. 6c) reduced food intake in goldfish at 1 hour post-injection. No significant changes in food intake were found in goldfish NLP injected fish at 2 and 4 hours post-injection (Fig. 6b). No effect on food intake was observed in response to an intraperitoneal injection of 100 ng/g body weight scrambled peptide (Fig. 6d), while rat NLP significantly reduced food intake (Fig. 6d).

### NLP Downregulates Preproghrelin and Orexin-A mRNA Expression and Upregulates CART mRNA Expression

**Figure 7:**
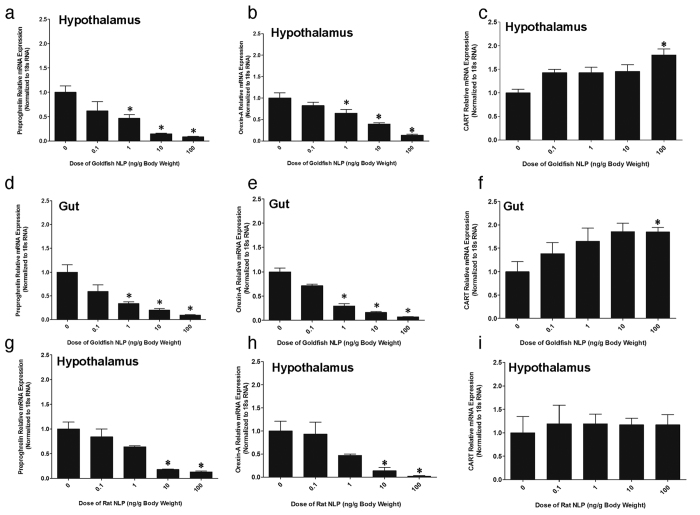
Goldfish NLP (1, 10, 100 ng/g BW) downregulated preproghrelin **(a)** and orexin-A **(b)** mRNA expression in the hypothalamus of goldfish when compared to saline treated control (denoted by 0), while upregulated CART mRNA expression at the highest dosage (100 ng/g BW) of NLP tested **(c)**. Similarly, goldfish NLP (1, 10, 100 ng/g BW) also reduced preproghrelin **(d)** and orexin-A **(e)** mRNA expression, and upregulated CART mRNA expression at the highest dosage of NLP (100 ng/g BW) in the gut of goldfish **(f)**. Intraperitoneal injection of rat NLP downregulated preproghrelin **(g)** and orexin-A **(h)** mRNA expression in the hypothalamus of goldfish. No significant change in CART mRNA expression in the hypothalamus of goldfish was found **(i)**. The mRNA expression data was normalized to 18 s RNA. Asterisks denote significant differences between control and NLP injected groups (*p < 0.05, n = 10 fish/group). Data are presented as mean + SEM. One-way ANOVA (non-parametric) followed by Tukey’s multiple comparison test was used for statistical analysis.

**Figure 8:**
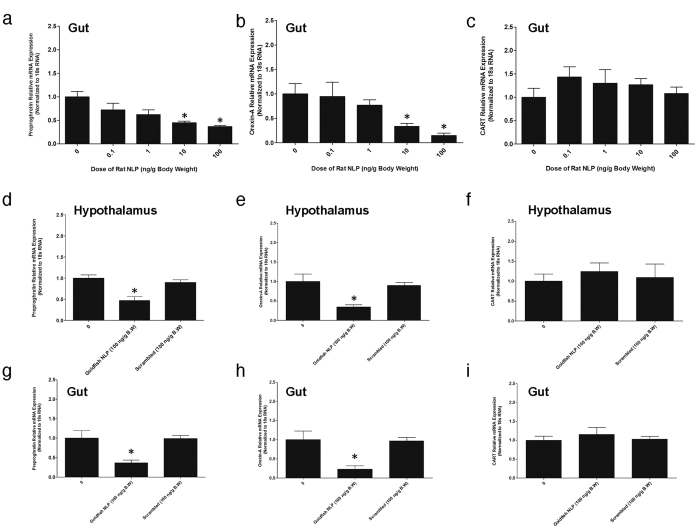
When compared to the saline control (denoted by 0), synthetic rat NLP (10, 100 ng/g BW) downregulated preproghrelin **(a)** and orexin-A **(b)** mRNA expression in the gut of goldfish. Rat NLP induced no changes in the expression of CART mRNA expression in the gut of goldfish **(c)**. NLP scrambled peptide did not elicit any significant effects on preproghrelin **(d,g)** or orexin-A **(e,h)** or CART **(f,i)** mRNA expression in the hypothalamus or gut. However, the intraperitoneal injection of goldfish NLP (100 ng/g) downregulated preproghrelin **(d,g)** and orexin-A **(e,h)** mRNA expression. The mRNA expression data was normalized to 18 s RNA. Asterisks denote significant differences (*p < 0.05) between control and NLP injected groups. One-way ANOVA followed by Tukey’s multiple comparison test was used for statistical analysis. Data are presented as mean + SEM. n = 6–10 fish/group.

Goldfish NLP (1, 10, 100 ng/g B.W) downregulated preproghrelin (Fig. 7a,d) and orexin-A (Fig. 7b,e) mRNA expression in hypothalamus and gut of goldfish. Goldfish NLP (100 ng/g B.W) also upregulated CART mRNA expression in the hypothalamus (Fig. 7c) and gut (Fig. 7f) of goldfish. Synthetic rat NLP (10 and 100 ng/g body weight) downregulated preproghrelin (Figs 7g and 8a) and orexin-A (Figs 7h and 8b) mRNA expression in the hypothalamus and gut. Rat NLP had no effect on CART mRNA expression (Figs 7i and 8c). No effect for the scrambled peptide was found on preproghrelin, orexin-A and CART mRNA expression (Fig. 8d–i).

## Discussion

We recently discovered that NUCB1 encoded NLP is insulinotropic in mice^16^. Our *in silico* analysis of NUCB1 sequences from different species found that NLP is very highly conserved among vertebrates. Here, we report the discovery of anorectic actions of NLP in fish, its tissue distribution and regulation of endogenous NUCB1. NLP sequence is conserved in zebrafish and goldfish, and shares high sequence identity with the M30 region of zebrafish NUCB2a and NUCB2b^13^. Previous research has shown that the M30 region of nesfatin-1 is critical for the satiety effects of nesfatin-1^6^. Considering the high sequence identity between the M30 region of nesfatin-1 and the corresponding region in NLP, it is highly likely that NLP has anorexigenic actions similar to nesfatin-1. NUCB1/NLP expression was abundant in the hypothalamus, hindbrain and midbrain while comparatively low expression was detected in other tissues. The results presented here are the first line of evidence for NUCB1 expression in the central nervous system of teleosts. These results are in line with previous studies that showed abundant NUCB2 expression in the appetite regulatory centers of hypothalamus in rats^5^ and goldfish^10^,^12^. Vast distribution of NUCB1 in fish tissues explains that NUCB1 might have central and peripheral effects. Our results indicate that hypothalamus is an abundant source of NUCB1 in goldfish suggesting a role for NLP in the central control of reproduction and metabolism.

To gain further understanding on the cell specific expression of NUCB1/NLP, we conducted immunofluorescence studies. NUCB1/NLP-like immunoreactivity was observed in the rostral pars distalis (RPD) region of goldfish anterior pituitary. RPD cells in goldfish are generally melanotrophs^12^. These observations are in agreement with previous results that indicated NUCB2/nesfatin-1-like immunoreactivity in the rostral pars distalis and pars intermedia (PI) in goldfish pituitary^12^. NUCB2/nesfatin-1 is also present in mice pituitary^17^. The presence of NUCB1/NLP-like ir in goldfish pituitary suggests a role for NUCB1 on pituitary hormone secretion. For example, nesfatin-1 regulates luteinizing hormone secretion in goldfish^12^. NUCB1/NLP-like ir was also detected in the mucosal cells lining the anterior intestine (J-loop). Previous studies have shown that NUCB2/nesfatin-1-like ir is found in the villi of goldfish gut and that nesfatin-1 suppresses ghrelin^11^. NUCB2/Nesfatin-1 like immunoreactivity was abundant in the glandular cells of gastrointestinal tract in rats^18^, and glucose stimulates its release^19^. It is likely that NUCB1 and NLP are also secreted in a meal-responsive manner from the gut. The current study also demonstrated the localization of NUCB1/NLP in goldfish reproductive organs. NUCB1/NLP-ir was observed in the ovarian thecal or follicular cells and in the leydig cells of goldfish testis, and was found colocalized with leydig cell marker SOX9^20^,^21^ and follicular and/or thecal cell marker FOXL2^20^,^21^. The results are in line with previous findings of NUCB2/nesfatin-1-ir within the follicular cells of goldfish and zebrafish ovaries^12^. Previous reports have showed the localization of NUCB2/Nesfatin-1-ir in leydig cells in Japanese quail^22^ and rats^23^. The localization of NUCB1/NLP-ir in the ovary and testis suggests a possible role for NLP in gonadal physiology and sex steroid hormone production. The extensive cell specific localization of NUCB1/NLP in goldfish suggests multiple biological functions for NLP in fish.

Next, we determined some factors that regulate the tissue specific expression of NUCB1 mRNA. While it was found that macronutrients fat, carbohydrate and protein suppress NUCB1 mRNA expression, these effects were highly tissue specific and were dependent on the duration of feeding. Although nutrient regulation of nucleobindins in fish is understudied, the results reported here are in agreement with previous research in mice, where high fat and protein diets elicited a reduction in NUCB2 mRNA^8^. This meal responsive inhibition of NUCB1 mRNA is suggestive of a role for NUCB1/NLP in fish feeding. It was also detected that NUCB1 mRNA displayed a daily rhythmic expression profile in the presence of LD cycle. NUCB1 mRNA in goldfish pituitary peaked during the light (ZT-8) and dark phases (ZT-16). In the gut, NUCB1 mRNA expression was lowest during the light phase when the fish feeding occurs, and relatively higher in the dark phase. These are the first results on a daily rhythmic profile of NUCB1/NLP expression in both central and peripheral tissues of a vertebrate. Estradiol treatment downregulated NUCB1 mRNA expression in goldfish pituitary, while testosterone upregulated NUCB1 expression in goldfish gut, hindbrain and hypothalamus. Chung *et al*.^17^ observed a significant increase in NUCB2 mRNA expression post estradiol administration in ovariectomized mice. The differences between the studies might be due to the use of ovariectomized mice, and/or species-specific differences in the interactions of gonadal steroids and NUCB1. Food deprivation is known to play a significant role in altering neuroendocrine factors that play critical role in reproduction and energy intake in several species^13^,^24^. In goldfish, fasting for 7 or 28 days did not result in any significant changes in body weight and this result is in agreement with previous reports in goldfish^25^,^26^,^27^. NUCB1 expression in gut was significantly lower when fish were food deprived for one week^13^. This is consistent with previous study on NUCB2 expression in goldfish gut and Ya fish that showed a significant downregulation after 7-day food deprivation^10^,^14^. Similar results were also reported in rodents, showing a decrease in NUCB2 expression in gastric endocrine cells^18^. The negative modulation of NUCB1 in response to fasting suggests anorexigenic actions for NUCB1 and/or encoded NLP. These new findings on endogenous NUCB1, especially the energy status and daily rhythm profile and the dependence of its expression provide new insights on NUCB1/NLP biology in fish.

Our next study determined if NLP is indeed biologically active in goldfish. When comparing the mid segment (M30) region of goldfish/zebrafish NLP and corresponding synthetic rat NLP region, it was found that both sequences have 80% amino acid sequence identity. Due to this very high sequence identity, we first injected goldfish with rat NLP. Intraperitoneal injection of synthetic rat NLP inhibited feed intake in goldfish by 40% and 68% at 10 and 100 ng/g body weight doses respectively over a period of 1 hour. Intraperitoneal injection of goldfish NLP also decreased feed intake by approximately 30% and 60% corresponding to similar doses over a period of 1 hour in goldfish. To confirm that the NLP elicited satiety effect requires a specific sequence, we injected goldfish with a scrambled peptide based on NLP. The scrambled peptide did not elicit any changes in food intake of goldfish. These data clearly indicate an anorexigenic action for NLP in goldfish. Since NLP had a profound anorexigenic action in goldfish, we explored whether NLP influences other appetite regulatory factors, including ghrelin, orexin-A and cocaine and amphetamine regulated transcript (CART) to elicit its satiety effects.

Ghrelin and orexin-A are potent orexigens in goldfish^28^,^29^, and CART, is an anorexigen^30^. Nesfatin-1 was found to suppress ghrelin and orexin-A in goldfish^11^. Similar to this, a significant downregulation of ghrelin and orexin-A mRNA expression by NLP (10 and 100 ng/g B.W) was observed in goldfish hypothalamus and gut. In contrast, NLP administration (100 ng/g body weight) upregulated CART mRNA expression in goldfish gut. IP injected NLP could possibly cross the blood brain barrier in a manner similar to nesfatin-1^31^,^32^ to induce satiety. These results show that NLP, similar to nesfatin-1 could suppress hormones in the orexigenic pathways and stimulate anorexigenic pathways to decrease food intake.

This research discovered several key aspects of NUCB1/NLP biology. First, it uncovered the satiety effects of NLP. Second, tissue and cell specific expression of NUCB1 was determined. Third, we found macronutrients, energy status, sex steroids and daily rhythms as four regulators of endogenous NUCB1. These results are of importance, and add significant new information to our growing knowledge on naturally occurring regulators of metabolic and endocrine functions in vertebrates. Our discoveries outlined here provide the first line of evidence on biological activity of NUCB1 encoded NLP in goldfish. The processing of endogenous NLP from NUCB1 and its mode of action are important new directions to consider during future investigations on nucleobindins and NLP.

## Materials and Methods

### Animals

Goldfish (*Carassius auratus*, common variety) were purchased from Aquatic Imports (Calgary, Canada). Goldfish (4–5 inches long, body weight: 25 g) were maintained at 24 °C under 12L:12D photoperiod cycle. Unless otherwise specified, fish were fed once daily with a 4% body weight ration at 11 AM with slow sinking pellets (slow-sinking pellets; Aqueon, Catalog # 06053). Euthanasia was conducted using 0.5% tricaine methanesulfonate-222 (TMS-222, Syndel Laboratories, BC, Canada) followed by spinal transection. All animal experimentations complied with the policies of the Canadian Council for Animal Care, and were approved by the University of Saskatchewan Animal Research Ethics Board (2012-0082).

### *In Silico* Analysis

NUCB1 sequences from various species were obtained from GenBank (http://www.ncbi.nlm.nih.gov/genbank/ ) and aligned using Clustal Omega (http://www.ebi.ac.uk/Tools/msa/clustalo/). The signal peptide site in the zebrafish NUCB1 sequence was predicted using SignalP 4.1 server (http://www.cbs.dtu.dk/services/SignalP/). The GenBank Accession numbers of sequences used are provided in figure legends.

### Relative mRNA expression of NUCB1 by Real-time Quantitative PCR (RT-qPCR)

Samples of brain, anterior intestine (J-loop region), midgut, rectum, eye, liver, heart, muscle, gill, testis, ovary and skin were collected from goldfish in order to study the tissue distribution of NUCB1 (n = 6). Fish were anesthetized using 0.5% tricaine methanesulfonate (TMS; Syndel Laboratories, Catalog # 5980A) before dissection. Tissue samples were collected for total RNA extraction and were stored in −80 °C. Total RNA was extracted using TRIzol RNA extraction reagent (Catalog # 15596-026, Invitrogen). cDNAs were synthesized using iScript cDNA reverse transcription supermix (Bio-Rad Laboratories, Catalog # 170-8841). The cDNAs were used for RT-qPCR. The primers used during this research were: Goldfish/zebrafish NUCB1 (sense primer 5′-CTGTCTCTGTGTCTGCTGGT-3′ and antisense primer 5′-TGGTGCTGTCCAGTTTAGCC -3′; annealing temperature- 60 °C), goldfish beta-actin (sense primer 5′-CTACTGGTATTGTGATGGACT-3′ and antisense primer 5′-TCCAGACAGAGTATTTGCGCT-3′; annealing temperature- 59 °C), goldfish 18s RNA (sense primer 5′-GGATGCCCTTAACTGGGTGT-3′ and antisense primer 5′-CTAGCGGCGCAATACGAATG-3′; annealing temperature- 60 °C), goldfish elongation factor-1α (EF-1α) (sense primer 5′-CCCTGGCCACAGAGATTTCA-3′ and antisense primer 5′-CAGCCTCGAACTCACCAACA-3′; annealing temperature- 60 °C), goldfish ghrelin (sense primer 5′-ATTCAGAGTGTTGTCGTA-3′ and antisense primer 5′-AGGAAAGAGCACATAAGA-3′; annealing temperature- 56.6 °C), goldfish CART (sense primer 5′-GTGCCGAGATGGACTTTGAC-3′ and antisense primer 5′-AGCTGCTTCTCGTTGGTCAG-3′; annealing temperature- 60 °C) and goldfish orexin-A (sense primer 5′-GCATATCGGCCGCTTTAATA-3′ and antisense primer 5′-GGGTCCTCGAGTCTCTTTCC; annealing temperature- 60 °C). The primers were validated and optimized for efficiency and annealing temperatures. Real-time quantitative PCR was carried out using iQ SYBR Green supermix (Bio-Rad, Catalog # 170-8880) and CFX Connect Optics module system (Bio-Rad, Canada) controlled by CFX Connect PC-based software (Bio-Rad, Canada) and was analyzed using the Livak method described earlier^11^. Relative mRNA expression of genes of interest were quantified and normalized to the expression of elongation factor 1α (EF1α) (tissue distribution), beta actin (diet study, daily rhythms) and 18s RNA (food deprivation, estradiol and testosterone treatment and food intake studies) were used as housekeeping genes in the respective studies performed during the research. The internal control gene was chosen based on the gene that provided the most consistent Ct values provided in each study.

### Western Blot Analyses

Goldfish brain, pituitary, gut, ovary and testis samples were collected to confirm the presence of NUCB1 by Western blot analysis, conducted as described earlier ref. 16. Fish (n = 4) were anesthetized using 0.5% TMS-222 before dissection. Tissues for Western blot were homogenized using T-PER tissue protein extraction reagent (Catalog # 78510, Thermo Scientific, Vantaa, Finland) followed by protein concentration determination by Bradford assay using NanoDrop 2000c (Thermo). The samples were prepared using 1X Laemmli buffer containing 0.2% of 2-mercaptoethanol (Bio-Rad, Catalog # 161-0737 and -0710) and the samples were subjected to boiling at 95 °C for 5 min followed by vortexing prior to loading. The whole sample volume (30 μL), each containing 40 μg protein was electrophoresed on a Mini-PROTEAN TGX 8–16% gradient gel (Bio-Rad, Catalog # 456-1104) at 200 V for 20–30 min. After the run, the proteins were transferred to a 0.2 μm BioTrace nitrocellulose membrane (PALL Life Sciences, Catalog # 27377-000) subjected to blocking using 1X RapidBlock solution (aMRESCO, Catalog # M325). In order to detect the presence of NUCB1 and vinculin (reference) protein, rabbit polyclonal anti-mouse nucleobindin-1 (custom synthesized, Catalog # 1312- PAC- 02, 1:3000, Pacific immunology, Ramona, CA) for NUCB1 and rabbit polyclonal anti-vinculin (Catalog # ab73412, 1:1000, Abcam, Massachusetts) for vinculin were used. As secondary antibody, goat anti-rabbit IgG (H + L) HRP conjugate (Catalog # 170-6515, 1∶3000, Bio-Rad) was used. For visualization of protein, the membrane was incubated for 5 min in Clarity Western ECL substrate (Bio-Rad, Catalog # 170-5061) and imaged using ChemiDoc MP imaging system (Bio-Rad, Catalog # 170-8280). Stripping for detection of reference protein in the membrane was conducted using western blot stripping buffer (Thermo Scientific, Catalog # 46430). Primary antibody was pre-absorbed in 3.33 μg synthetic goldfish/zebrafish NLP (Pacific immunology, Ramona, CA) overnight and was used as pre-absorption controls for goldfish tissues to confirm the specificity of the NUCB1 antibody. Precision plus protein dual Xtra standards (Bio-Rad, Catalog # 161-0377) were used as markers to detect the molecular weight of protein of interest (NUCB1, preabsorption and vinculin). The NUCB1 antibody used in this study detects both the precursor NUCB1 and processed NLP.

### Immunohistochemistry

The presence and localization of the protein in different tissue sections of goldfish were detected by immunohistochemical (IHC) studies. For IHC studies conducted as described in detail earlier^16^,^26^, pituitary, ovary, J-loop and testis were collected from goldfish. The antibodies used were: rabbit polyclonal anti- mouse nucleobindin-1 (Pacific immunology, Ramona, CA) for NUCB1, mouse monoclonal anti-growth hormone (Catalog # CLX 130AP, 1:500, Cedarlane, USA) for growth hormone in pituitary, mouse monoclonal anti-ghrelin hormone (Catalog # ab57222, 1:500, Abcam, Massachusetts) for ghrelin in J-Loop^11^, mouse monoclonal anti-SOX9 hormone (Catalog # ab76997, 1:500, Abcam, Massachusetts) for SOX9 in testis^20^,^33^, goat monoclonal anti-FOXL2 hormone (Catalog # PA5-18175, 1:500, Waltham, MA ) for FOXL2^20^,^33^ staining in ovary respectively. The ghrelin antibody was previously validated for use in goldfish^11^. Both NUCB1 and GH antibodies were validated in this research using preabsorption controls. SOX9 and FOXL2 antibodies were raised against an epitope that has approximately 85% similarity with zebrafish SOX9 and FOXL2. The slides were then washed with PBS and then were incubated with secondary antibody for one hour at room temperature. Goat polyclonal anti-rabbit IgG (Catalog # TI-1000, 1:500 dilution, Vector Laboratories, California) for NUCB1, goat polyclonal anti-mouse IgG H&L (FITC green- Growth Hormone, ghrelin, SOX9, Catalog # ab6785, 1:500 dilution, Abcam, Massachusetts) and Donkey Anti-Goat IgG H&L (Alexa Fluor 488, FOXL2; Catalog # ab150129, 1:500 dilution, Abcam, Massachusetts) were used as secondary antibodies. The slides were then rewashed with PBS and were mounted on Vectashield medium containing DAPI dye (Blue, Vector Laboratories). The slides were dried and imaged using a Nikon inverted microscope (L100) (Nikon DS-Qi1 MC camera, ON, Canada) and analyzed using NiS Elements imaging software (Nikon, Canada). Slides incubated with secondary antibody alone, or preabsorption^11^ using synthetic goldfish NLP were used as negative controls. Since the antibody used here detects both NUCB1 and NLP, we used the term NUCB1/NLP-like immunoreactivity to refer to the staining obtained in our immunohistochemical studies.

### NUCB1 Tissue Expression Profile in Goldfish

Goldfish (n = 6 fish/group) were maintained and fed daily at Zeitgeber time- 4 (ZT-4) as described earlier^21^. On the day of study, fish were collected randomly and sampling was done at 4 hour intervals throughout the 24-h cycle period starting from ZT-0 (light phase) until ZT-24 (dark phase). On the day of experiment fish were fed at ZT-4 respectively. Upon euthanasia, fish were dissected and hypothalamus, gut, hindbrain, forebrain and pituitary were collected and stored at −80 °C until total RNA extraction. Sampling of tissues during the dark phase was done under dim red lighting. To study the relative mRNA expression of NUCB1, RT-qPCR studies were carried out as described earlier on hypothalamus, gut, forebrain, hindbrain and pituitary of goldfish.

### *In Vivo* Diet Study

Goldfish were weight matched (n = 8/group) and five groups of fish were fed with five different diets (custom diet containing high carbohydrate, high protein, high fat (9%), very high fat (20%) and control) for 1 and 4 weeks. The details of diet composition are provided in Blanco *et al*.^34^. The calorie content of the respective diets were: control (TestDiet, Catalog # 8887) 3.43 kcal/g^2^ with 37.6% energy derived from protein, 46.6% energy derived from carbohydrate and 15.8% energy derived from fat; high carbohydrate (TestDiet, Catalog # 8890) 3.87 kcal/g^2^ with 29.2% energy derived from protein, 56.8% energy derived from carbohydrate and 14% energy derived from fat; high protein (TestDiet, Catalog # 8893) 3.59 kcal/g^2^ with 50.4% energy derived from protein, 44.5% energy derived from carbohydrate and 5.1% energy derived from fat; high fat (9%) (TestDiet, Catalog # 8889) 3.61 kcal/g^2^ with 35% energy derived from protein, 49.9% energy derived from carbohydrate and 15.1% energy derived from fat; very high fat (20%) (TestDiet, Catalog # 8886) 4.27 kcal/g^2^ with 30.3% energy derived from protein, 27.6% energy derived from carbohydrate and 42.1% energy derived from fat. Hypothalamus and gut were collected upon euthanasia, followed by measurement of NUCB1 mRNA expression relative to the expression of beta-actin as reference gene.

### *In Vivo* Food Deprivation Studies

In this study, the expression of NUCB1 mRNA was determined upon food deprivation in goldfish. The relative expression of NUCB1 was determined in goldfish hypothalamus and gut after 3 and 7 days of food deprivation (n = 6/group). Fish in the control group were fed as usual. On the sampling day (3 or 7 days), during 11 AM–12 PM, goldfish from the fed and unfed cohorts were euthanized using 0.5% TMS-222 post 1 hour of feeding time. Subsequently, the hypothalamus and gut tissues were collected and stored at −80 °C for total RNA extraction followed by measurement of NUCB1 mRNA expression relative to expression of 18 s RNA housekeeping gene.

### *In vivo* Treatment of Goldfish with Estradiol and Testosterone

For this study, female goldfish (n = 7/tank) were maintained as described earlier. On the day of experiment, solid silicone pellets containing estradiol or testosterone were prepared and washed thoroughly in saline and implanted intraperitoneally. Details of this study were previously validated in our lab^35^. Three different doses 25, 50 and 100 μg/g BW were initially considered and compared to no treatment group (control). We found that 100 μg/g BW of estradiol or testosterone was effective in elevating steroid hormone levels in goldfish and this dose was used to study the NUCB1 mRNA expression in goldfish. After 2.5 days of implantation, fish were euthanized and forebrain, hindbrain, gut, hypothalamus, pituitary were collected and NUCB1 relative mRNA were quantified using RT-qPCR.

### Effect of Exogenous NLP on Feed intake and Appetite Regulatory Peptides in Goldfish

Fish (n = 6/group in each study) were maintained as described earlier. Synthetic rat and goldfish/zebrafish NLP were intraperitoneally injected (200 microliters) at four different doses i.e. 0, 0.1, 1, 10 and 100 ng/g BW just prior to their scheduled feeding time (11 AM). Goldfish/Zebrafish NLP (VPIDRNPDPPQEEKAEENVDTGLYYDRYLREVIEVLETDPHFREKLQTANTEDIKNGRLSKELDLVGHHVRTRLDEL) was synthesized by Pacific immunology (Ramona, CA), and synthetic rat NLP (VPVDRAAPHQEDNQATETPDTGLYYHRYLQEVINVLETDGHFREKLQAANAEDIKSGKLSQELDFVSHNVRTKLDEL) was synthesized by Abgent Technologies, California with >95% purity. A scrambled peptide (79 amino acids) was designed using the Sequence Manipulation Suite™ online tool (www.bioinformatics.org/sms2/). NLP Scramble peptide (PDSRSDDGSPSVQLQDYALIADAEVTLTHIELFGSPQNATKLLNKTERLRFLKVVRGKHRENVVATEHYQAQKYPEEDE) with the lowest similarity to the rat NLP sequence was selected. The peptide synthesized was >95% pure (Pacific Immunology Corp, California, USA) and the mass and purity were confirmed by LC-MS. The control group (n = 6) were injected with 0.9% sodium chloride. Immediately after NLP administration fish were allowed to recover and were briefly fed and the feed was recovered post 1 hour (dried overnight at 60 °C) to quantify feed intake. In the time course study, food was recovered at 1, 2 or 4 hours post-injection. Goldfish euthanasia, tissue collection and processing and RT-qPCR were conducted as described earlier. Tissues collected were used for studying the expression of appetite regulatory peptides preproghrelin, orexin-A and CART mRNAs. Data were normalized to 18 s RNA (housekeeping gene).

### Statistical Analysis

Data were analyzed using one-way ANOVA (non-parametric tests) followed by Tukey’s multiple comparison test, or Tukey Kramer’s test. PRISM version 5 (GraphPad Inc., USA) and IBM SPSS version 21 (IBM, USA) were used for statistical analysis. P < 0.05 was considered statistically significant. Data are represented as mean + SEM. For daily rhythmicity of NUCB1/NLP gene expression, cosinor analysis was used by fitting periodic sinusoidal values in relevance to the expression values of the gene of interest for the seven time points taken into consideration during the study. Cosinor analysis was performed using the formula f(t) = M + Acos(tπ/12−ϕ), where f(t) is the gene expression level in a given time, the mesor (M) is the mean value, A is the sinusoidal amplitude of oscillation, t is time in hours and ϕ is the acrophase (time of peak expression). Significance of cosinor analysis was analyzed using the zero-amplitude test, which specifies that if sinusoidal amplitude differs from 0 with a given probability during the 24-h profile. The time series data were plotted to display a 24-h rhythmic pattern with cosinor analysis.

## Additional Information

**How to cite this article**: Sundarrajan, L. *et al*. Nesfatin-1-Like Peptide Encoded in Nucleobindin-1 in Goldfish is a Novel Anorexigen Modulated by Sex Steroids, Macronutrients and Daily Rhythm. *Sci. Rep.* **6**, 28377; doi: 10.1038/srep28377 (2016).

## Supplementary Material

Supplementary Information
