# Supplementary material for: Nesfatin-1-Like Peptide Encoded in Nucleobindin-1 in Goldfish is a Novel Anorexigen Modulated by Sex Steroids, Macronutrients and Daily Rhythm
Source: Sci Rep. 2016 Jun 22;6:28377. doi: 10.1038/srep28377 (PMC4916606; doi:10.1038/srep28377)

Nesfatin-1-Like Peptide Encoded in Nucleobindin-1 in Goldfish is a Novel Anorexigen Modulated by Sex Steroids, Macronutrients and Daily Rhythm.

Lakshminarasimhan Sundarrajan, Ayelén Melisa Blanco, Juan Ignacio Bertucci,
Naresh Ramesh, Luis Fabian Canosa, Suraj Unniappan

Supplementary Figure 1 – Title page

Supplementary Figure 1: Western blot analysis showing the specificity of the NUCB1 antibody used for Western blot analysis and immunohistochemistry. In figure a below, it is shown that the antibody detects only synthetic NLP, but not nesfatin-1. In the figure b, the NLP staining disappeared when the antibody was pre-absorbed with goldfish synthetic NLP.

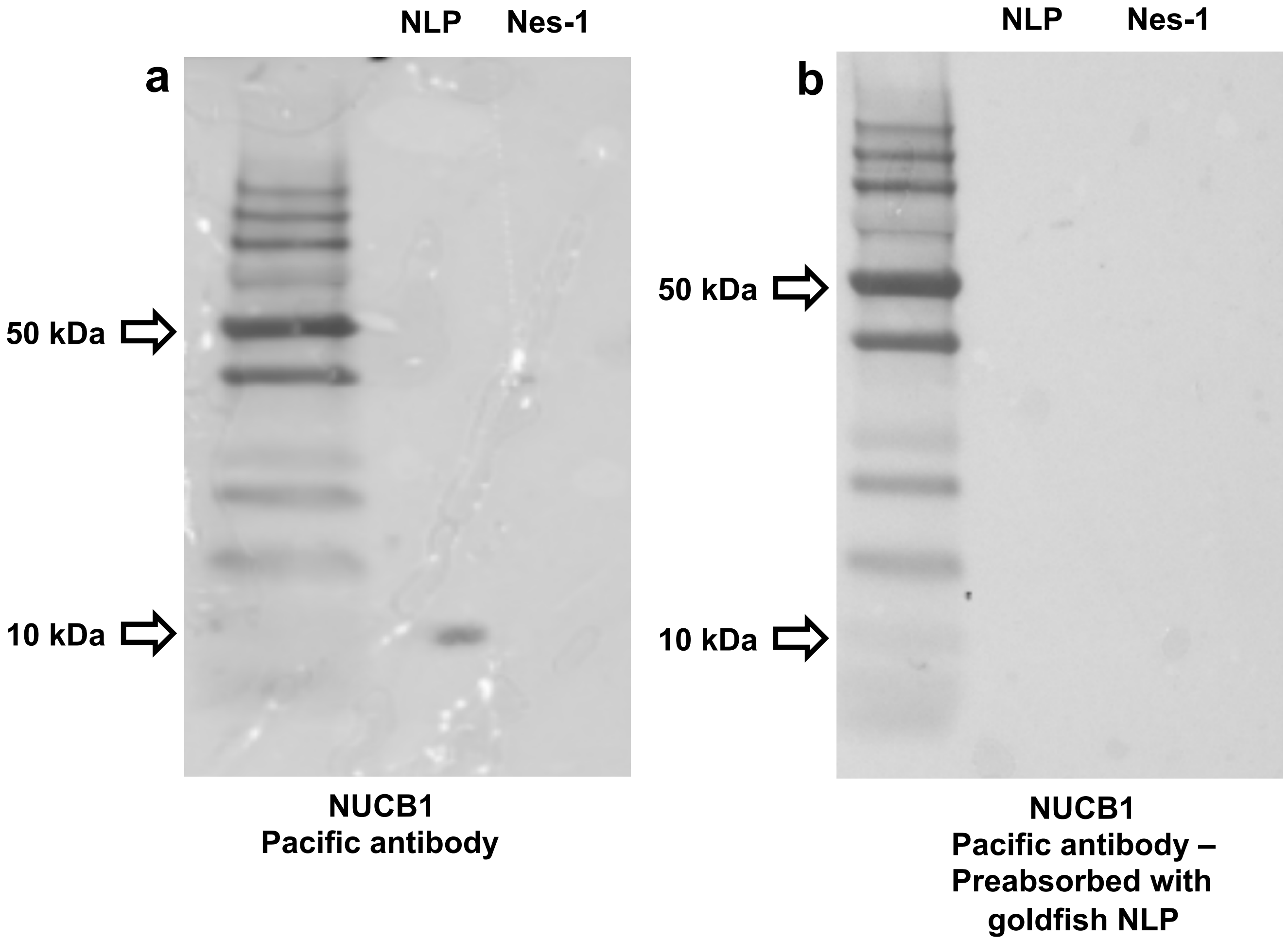

Supplement: Supplementary Information [file srep28377-s1.pdf]
